# Endoscopic Submucosal Dissection for Early Colorectal Neoplasms: Clinical Experience in a Tertiary Medical Center in Taiwan

**DOI:** 10.1155/2013/891565

**Published:** 2013-02-25

**Authors:** Mei-Yu Tseng, Jung-Chun Lin, Tien-Yu Huang, Yu-Lueng Shih, Heng-Cheng Chu, Wei-Kuo Chang, Tsai-Yuan Hsieh, Peng-Jen Chen

**Affiliations:** Division of Gastroenterology, Tri-Service General Hospital, National Defence Medical Center, Neihu, Taipei 114, Taiwan

## Abstract

*Objectives*. Endoscopic submucosal dissection (ESD) is a promising technique to treat early colorectal neoplasms by facilitating en bloc resection without size limitations. Although ESD for early gastrointestinal epithelial neoplasms has been popular in Japan, clinical experience with colorectal ESD has been rarely reported in Taiwan. *Methods*. From March 2006 to December 2011, 92 consecutive patients with early colorectal neoplasms resected by ESD at Tri-Service General Hospital were included. ESD was performed for colorectal epithelial neoplasms with a noninvasive pit pattern which had the following criteria: (1) lesions difficult to remove en bloc with a snare, such as laterally spreading tumors-nongranular type (LST-NG) ≧20 mm and laterally spreading tumors-granular type (LST-G) ≧30 mm; (2) lesions with fibrosis or which had recurred after endoscopic mucosal resection with a nonlifting sign. *Results*. The mean age of the patients was 66.3 ± 12.9 years, and the male-female ratio was 1.8 : 1. The mean tumor size was 37.2 ± 17.9 mm. The en bloc resection rate was 90.2% and the R0 resection rate was 89.1%. Perforations during ESD occurred in 11 patients (12.0%) and all of them were effectively treated by endoscopic closure with hemoclips. No delayed perforation or postoperative bleeding was recorded. There were no procedure-related morbidities or mortalities. *Conclusion*. ESD is an effective method for en bloc resection of large early colorectal neoplasms and those with a nonlifting sign. An endoscopic technique to close perforations is essential for colorectal ESD.

## 1. Introduction

Colorectal cancer (CRC) is one of the most common malignancies worldwide and is currently the third leading cause of cancer death in Taiwan [1]. Traditionally, surgical resection is the treatment of choice for large CRCs [2]. However, endoscopic submucosal dissection (ESD) has been widely accepted as a standard method for the treatment of large early colorectal malignant lesions in Japan. Several studies have shown encouraging results with the technique and it can provide a high accuracy of histological diagnosis without an increased local recurrence rate [3–7]. However, there are some imitations to the application of colorectal ESD, including the technical difficulty and a higher rate of complications and longer procedure time than with endoscopic mucosal resection (EMR).

There have been no reports on ESD for early colorectal neoplasms in Taiwan. To further enhance the clinician's understanding of the usefulness of this procedure and its related risks, we report our experience in performing ESD for early colorectal neoplasia in a medical center in Taiwan. The current practice and clinical outcome of ESD were also analyzed.

## 2. Patients and Methods

**Figure 1:**
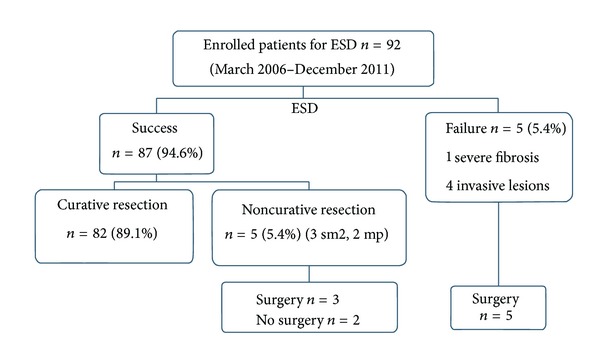
Patients and ESD outcomes. ESD: endoscopic submucosal dissection; sm2: submucosal invasion ≥1000 *μ*m; mp: muscularis propriae invasion.

From March 2006 to December 2011, 92 consecutive patients with colorectal neoplasms were treated with ESD at Tri-Service General Hospital (Figure 1). At present, the most critical factor in deciding whether to perform ESD is the probability of unexpected lymph node metastasis. The Paris classification and Kudo's pit pattern classification allow the prediction of the depth of invasion of early epithelial neoplasms [8, 9]. ESD was performed for colorectal neoplasms with a noninvasive pit pattern which had the following criteria [10]: (1) lesions difficult to remove en bloc with a snare, such as laterally spreading tumors-nongranular type (LST-NG) ≧20 mm, and laterally spreading tumors-granular type (LST-G) 226730 mm; (2) lesions with fibrosis or lesions which had recurred after EMR with a nonlifting sign.

For submucosal tumors, we also enrolled patients with symptomatic giant lipomas and rectal carcinoid tumors less than 10 mm that did not show regional lymph node enlargement on CT scanning or endoscopic ultrasonography. Rectal carcinoid tumors with distant metastases to the liver or lung on CT scans were excluded. Tumor locations were divided along cecum, right colon, left colon, and rectum. Right colon consists of ascending colon, hepatic flexure, and transverse colon. Left colon consists of the splenic flexure, descending colon, sigmoid colon, and rectosigmoid junction.

All patients were given a detailed explanation of the process and informed of potential complications of the procedure, including bleeding, perforation, and the possibility of additional surgery because of complications or histological diagnosis of resected specimens. Written informed consent was obtained. The ESD procedure was performed in accordance with the ethical principles of the Declaration of Helsinki. Our main outcome measurements included the en bloc resection rate, curative resection rate, procedural time, and complications.

### 2.1. ESD Procedures

**Figure 2:**
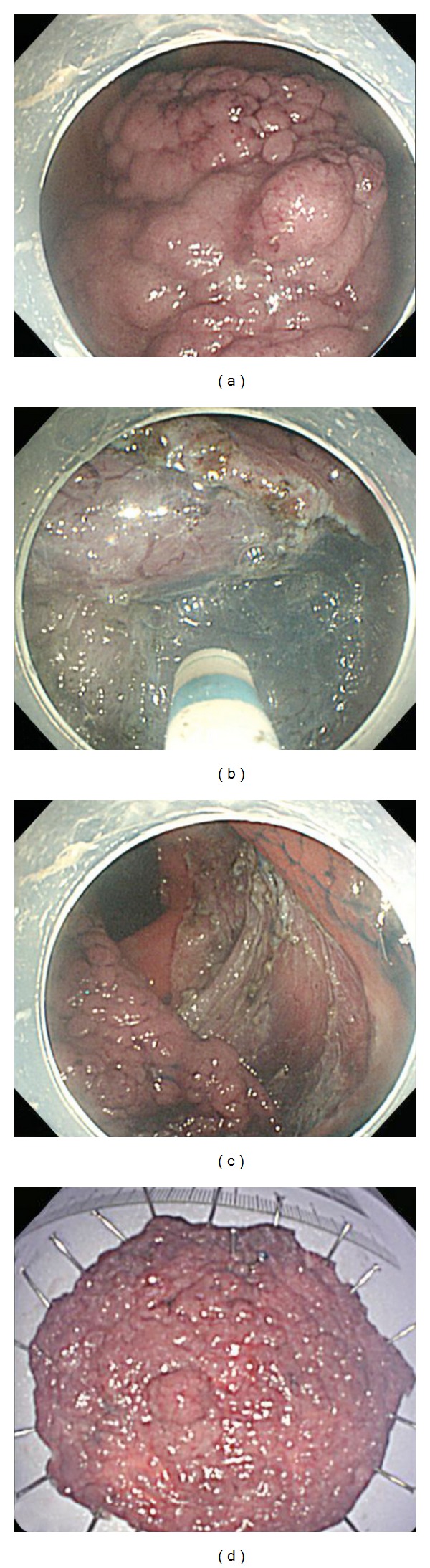
Procedure for colorectal ESD. (a) A large laterally spreading tumor-granular type is seen in the ascending colon. (b) Hyaluronic acid solution is injected into the submucosa, and the submucosal layer under the lesion is directly dissected from the underlying muscularis propria using lateral movement of the dual knife. (c) A huge artificial ulcer is created by ESD. (d) The 80 × 65 mm specimen is completely resected in one piece. ESD: endoscopic submucosal dissection.

All colon lesions were identified using high-magnification endoscopy (CF-H260AZI, PCF-Q260AZI; Olympus) with narrow-band imaging, and lesions were delineated with 0.4% indigo carmine spray dye (Figure 2(a)). ESD was principally carried out by using a single-channel endoscope with a Waterjet System (PCF-Q260AZI; Olympus). After injection of 10% glycerol, sodium hyaluronate acid 0.4% mixed with a small amount of indigo carmine dye and epinephrine was injected into the submucosal layer. The submucosal layer under the lesion was directly dissected from the underlying muscularis propria, using a lateral movement of the dual knife (KD 650U; Olympus) (Figure 2(b)). An artificial ulcer was created by ESD (Figure 2(c)). Carbon dioxide insufflation (UCR; Olympus) was used during colorectal ESD to reduce patient discomfort. Conscious sedation was achieved with a small amount of midazolam and pethidine if necessary.

### 2.2. Definition of Complications

Perforation during an ESD procedure was defined as immediate and delayed perforation as occurring after completion of the procedure. It included free air in the abdomen on image studies and tiny deep holes with visible omentum or other tissue outside the muscle layer, such as transparent serosa, on the endoscopic monitor. Procedure-related bleeding was defined as clinical evidence of hemorrhage with melena or hematochezia or that which required a special hemostatic method after the procedure. Procedure time was defined as the time from incision with the dual knife to the completion of removal the lesion. 

### 2.3. Histological Assessment

The excised specimens were stretched, pinned, and fixed in 10% buffered formalin for histological diagnosis (Figure 2(d)). An R0 resection was complete when histological examination revealed tumor-free lateral and vertical margins. En bloc resection was defined as resection in one piece of tissue. When submucosal invasion depth is 1,000 *μ*m or deeper than 1,000 *μ*m, we use the definition “massive submucosal invasion” [11]. When the tumor showed massive submucosal invasion or had unfavorable histological risk factors related to lymph node metastasis (i.e., lymphovascular invasion, poor differentiation, or tumor budding), we recommended surgical resection accompanied with lymph node dissection [12]. A curative resection was achieved when both the lateral and vertical margins of the specimen were free of cancer, and there was no SM invasion deeper than 1,000 *μ*m, or unfavorable histological risk factors related to lymph node metastasis.

### 2.4. Followup

Follow-up colonoscopy was performed every 6 months during the first year after ESD, and then annually thereafter. A biopsy was performed for histological assessment of any suspicious abnormality.

### 2.5. Statistical Analysis

Patients with and without complications during ESD procedures were compared using the independent two-sample *t*-test for continuous variables. Fisher's exact test was used for categorical variables; the data are presented as numbers (percentages). Logistic regression analysis was performed to analyze the odds ratios of significant factors associated with patients with complications. Linear regression analysis was used to determine factors correlated with the ESD procedure time. All baseline characteristics were evaluated as possible predictors, and those with *P* < 0.2 were included in the univariate and multivariate regression models. All statistical assessments were two-sided and differences were evaluated at the 0.05 level of significance. Statistical analyses were performed using SPSS 15.0 software (SPSS Inc., Chicago, IL, USA)

## 3. Results

### 3.1. Characteristics of the Subjects

The mean age of the 92 subjects was 66.3 ± 12.9 years (range 38–94 years), the male-to-female ratio was 1.8 : 1, and the mean tumor size was 37.2 ± 17.9 mm. The mean hospital stay for ESD was 4.0 ± 5.2 days. Eight tumors were located in the cecum (8.7%), 44 in the right side of the colon (47.8%), 17 in the left side of the colon (18.5%), and 23 in the rectum (25.0%). Macroscopic types included 35 LST-NG (38.0%), 50 LST-G (54.3%), and 7 submucosal tumors (7.6%). Histologically, there were 39 adenomas (42.4%), 32 intramucosal adenocarcinomas (34.8%), 5 adenocarcinomas with superficial submucosal invasion (<1000 *μ*m, 5.4%), 5 adenocarcinomas with deep submucosal invasion (5.4%), 4 adenocarcinomas with muscle layer invasion (4.4%), 5 carcinoid tumors (5.4%), and 2 lipomas (2.2%) (Table 1).

Immediate perforation occurred in 11 patients (12.0%) during the ESD procedure, all of which were treated successfully with hemoclips (HX-610-090L; Olympus). There was no significant postoperative hemorrhage or procedure-related mortality in our study. There were no significant differences in age, gender, tumor size, macroscopic type, or histology (Table 1).

In Table 2, the univariate logistic regression model indicated that tumor location and nonlifting sign were associated with complications (*P* < 0.05). Multivariate logistic regression indicated that tumor size (OR: 13.32, 95% CI: 1.22–146.02, *P* = 0.034), cecum (OR: 17.22, 95% CI: 1.25–236.78, *P* = 0.033), and nonlifting sign (OR: 5.97, 95% CI: 1.09, 32.63, *P* = 0.039) were associated with complications.

The mean procedure time was 59.0 ± 36.7 minutes. Univariate analysis in Table 3 shows that there were several factors associated with a longer procedure time, including tumor size, tumor location, and histology of the tumor. However, multivariate linear regression indicated that tumor size was the key factor for the procedure time (*P* = 0.001). The median follow-up duration was 27 months (range 11–79 months). No patients had local recurrence or distant metastasis after ESD.

## 4. Discussion

EMR is well established for the endoscopic removal of colorectal epithelial neoplasms with a flat morphology. To resect lesions larger than 20 mm in diameter, EMR leads to piecemeal resection. Colorectal ESD can overcome this disadvantage. This is the first large case series of colorectal ESDs performed at a specialized center in Taiwan.

After piecemeal resection, histological assessment is mostly impossible and the risk of incomplete resection or recurrence increases. Recently published studies on piecemeal EMR of large colorectal lesions (diameter >20 mm) have reported recurrence rates from 10% to 20.1% [17]. ESD can overcome this problem by allowing en bloc resection regardless of lesion size. Large studies from Japan have shown not only some advantages of colorectal ESD (a high en bloc resection rate even with large lesions, low recurrence rate) but also some disadvantages (time-consuming procedure, risk of complications) [18].

In the single-center study presented here, 92 ESD procedures in large lesions of the colorectum have been described. Procedures were performed over a 5-year period by one experienced endoscopist. A total of 110 gastric and esophageal ESD procedures were performed during the study period, reflecting the endoscopists' ESD expertise. The en bloc and R0 en bloc resection rates were 90.2% and 89.1%, respectively (Table 4). In this series, no local recurrence was recorded after ESD, with a median followup of 27 months (range 11–79 months).

Piecemeal resection of submucosal cancer lesions prevents reliable determination of the status of the resection margins. En bloc resection has the greatest benefit in histological evaluation of R0 resection, especially for lesions suspected of being malignant. If histological diagnosis discloses that submucosal invasion is less than 1000 *μ*m, without lymphovascular invasion or poor grading, the risk of lymph node metastasis is very low and surgery can be voided [19]. The risk of malignancy can be predicted by magnifying chromoendoscopy according to the Kudo pit classification. Also, the depth of submucosal invasion can be predicted with or without the invasive pattern [20]. In our study, en bloc resection was achieved in 90.2% of patients, and an accurate histological diagnosis could be made. Surgery was also suggested for those patients with submucosal invasion of more than 1000 *μ*m or unfavorable histological risk factors related to lymph node metastasis (i.e., lymphovascular invasion, poor differentiation, or tumor budding).

ESD can be performed not only for colorectal epithelial tumors but also for submucosal tumors. We performed ESD for 7 submucosal tumors consisting of carcinoid tumors and giant lipoma. In rectal carcinoid tumors smaller than 10 mm, lymph node metastasis was rare. Thus, it could be treated with colonoscopic resection. Complete resection of rectal carcinoid tumors is difficult with conventional EMR because these tumors originate from the submucosa. ESD is an effective treatment for submucosal tumors with dissection of the tumors directly along the submucosal layer and clear visualization of the submucosal resection margins. Several studies have reported better results with ESD compared with EMR for the treatment of rectal carcinoid tumors [7]. ESD may be considered for treatment of rectal carcinoid tumors because the technique shows a better histologically complete resection rate than that of EMR, has a low complication rate, and can be performed within a reasonable timeframe [21]. Kim et al. reported that the histologically complete resection rate of ESMR-L (endoscopic submucosal resection with a ligation device) of 93.3% is higher than the rates reported using various other conventional endoscopic resection methods (38%–90%) [22]. The procedure time was longer in the ESD group than in the ESMR-L group.

In this study, the mean diameter of the resected tumors obtained was 37 mm. In our case series of colorectal ESD, a high en bloc resection rate and low recurrence rate were obtained. However, the rate of perforations was still quite high (12.0%). In this study, perforations included free air in the abdomen on image studies and tiny deep holes with visible omentum or other tissue outside the muscle layer, such as transparent serosa, on the endoscopic monitor. The latter might not be true perforations but impending ones. In our study, tumor size, cecum location, and nonlifting sign were associated with perforations. ESD in cecum is risky because of its thin wall and concave structure. Perforations caused by ESD with the dual knife were small, about 1-2 mm in size, and could be closed with hemoclips immediately. All perforations were treated by endoscopic closure and medications, without surgery. A perforation rate is slightly higher than the rates from the other previous Japanese reports (2.3%–10.4%) [6].

In our study, a nonlifting sign was disclosed in 20.7% of all ESD lesions and was also a risk factor for perforation. For these lesions, it is technically impossible or risky to perform conventional EMR. Before the advent of ESD, patients with nonlifting intraepithelial neoplasms diagnosed endoscopically with a suspicion of malignancy were referred to a surgeon, and lesions which were unlikely to be malignant were treated by endoscopic ablation. ESD might be used as an alternative for the former lesions as a promising organ-preserving treatment. However, because of the relatively high perforation rate, the technique should be refined before it becomes a standard procedure.

Univariate and multivariate analyses revealed that large tumor size was an independent risk factor for operative time. ESD is a relatively long procedure, considerably longer than conventional EMR [18]. However, if a CO_2_ insufflation system is used, patients feel less discomfort and sometimes none at all during long procedures. These factors make the long procedure time for ESD less significant.

ESD is more difficult to perform in the colorectum than in the stomach because of anatomical features (thin wall, peristalsis, and folds). The perforation risk in colonic ESD is reported to be higher and endoscopic closure of colonic perforations seems to be more difficult than in the stomach [5]. However, colorectal ESD shows a clear learning curve, with decreasing procedure times and decreasing perforation rates over time [23]. Because of its technical difficulty, complication risks, and the learning curve, the role of ESD in colorectal lesions is not defined in countries outside Japan, and EMR is currently the standard treatment. The ESD operator in this study had performed more than 30 cases of gastric ESD before starting colorectal ESD. To perform colorectal ESD, with these experiences of gastric ESD, the learning curve and dissection speed of this operator in the early period were similar to those in the late period. The mean procedure time was 59.0 minutes in the current study, similar to the Japanese data, which report median procedure times of 70–116 minutes [3, 4, 18]. 

The limitations of this study include the fact that it was not performed as a randomized trial comparing ESD and EMR. Prospective randomized trials comparing these two procedures are needed in Taiwan to determine ESD indications for different types of lesions. Another limitation of this study is that no long-term outcome data are yet available, because only a few institutions have started performing colorectal ESDs in recent years. 

In conclusion, this study showed that ESD is a promising technique for the resection of early colorectal neoplasms. ESD performed by experienced endoscopists is a safe and very effective procedure for treating large superficial colorectal tumors and neoplastic lesions with a nonlifting sign. Since the complication rate is not low, the benefits and underlying risks might be different depending on the tumor characteristics and the endoscopist's skill. Further studies in Taiwan are needed to confirm our results for colorectal ESD.

## Figures and Tables

**Table 1:** Patient characteristics (*n* = 92).

	Total (*n* = 92)
Age (years)	66.3 ± 12.9
Gender, *n* (%)	
Male	59 (64.1)
Female	33 (35.9)
Tumor size (mm)	37.2 ± 17.9
Tumor location, *n* (%)	
Cecum	8 (8.7)
Right colon	44 (47.8)
Left colon	17 (18.5)
Rectum	23 (25.0)
Macroscopic type, *n* (%)	
LST-NG	35 (38.0)
LST-G	50 (54.3)
Submucosal tumor	7 (7.6)
Histology, *n* (%)	
Carcinoid tumor	5 (5.4)
Lipoma	2 (2.2)
Adenoma, high grade dysplasia	39 (42.4)
Adenocarcinoma sm1	5 (5.4)
Adenocarcinoma sm2	5 (5.4)
Adenocarcinoma mp	4 (4.4)
Adenocarcinoma m	32 (34.8)
Nonlifting sign, *n* (%)	
Negative	73 (79.3)
Positive	19 (20.7)
Previous biopsy, *n* (%)	
Negative	63 (68.5)
Positive	29 (31.5)
Procedure time (minutes)	59.0 ± 36.7

m: intramucosal cancer; sm1: submucosal invasion <1000 *μ*m; sm2: submucosal invasion ≥1000 *μ*m; mp: muscularis propriae invasion; LST-NG: laterally spreading tumors-nongranular type; LST-G: laterally spreading tumors-granular type.

**Table 2:** Univariate and multivariate analyses of factors affecting perforation in ESD (*n* = 92).

	Univariate		Multivariate	
	OR (95% CI)	*P* value	OR (95% CI)	*P* value
Age (years)	1.03 (0.98, 1.08)	0.278		
Gender				
Female	1.00		1.00	
Male	1.16 (0.85, 1.60)	0.351		
Tumor size (mm)	6.53 (0.80, 53.51)	0.080	13.32 (1.22, 146.02)	0.034
Tumor location				
Cecum	13.2 (1.13, 154.92)	0.040	17.22 (1.25, 236.78)	0.033
Right colon	2.20 (0.23, 20.92)	0.493	1.05 (0.04, 11.68)	0.968
Left colon	4.71 (0.45, 49.94)	0.198	5.65 (0.46, 69.08)	0.175
Rectum	1.00		1.00	
Macroscopic type				
LST-NG	1.00			
LST-G	0.38 (0.10, 1.39)	0.143		
Histology				
Adenocarcinoma	1.00			
Adenoma	1.46 (0.41, 5.20)	0.564		
Nonlifting sign, *n* (%)				
Negative	1.00		1.00	
Positive	3.51 (1.65, 9.71)	0.040	5.97 (1.09, 32.63)	0.039
Procedure time	1.01 (0.99, 1.02)	0.352		

ESD: endoscopic submucosal dissection; LST-NG: laterally spreading tumors-nongranular type; LST-G: laterally spreading tumors-granular type.

**Table 3:** Univariate and multivariate analysis of factors affecting ESD procedure time.

	Univariate		Multivariate	
	*β* ± SE	*P*	*β* ± SE	*P*
Age	0.41 ± 0.30	0.171	0.19 ± 0.27	0.50
Gender (male)	−10.91 ± 7.94	0.173	−11.88 ± 7.07	0.10
Tumor size	8.85 ± 1.96	<0.001	7.00 ± 2.04	0.001
Tumor location (rectum)				
Cecum	36.41 ± 14.71	0.015	17.08 ± 12.54	0.18
Right colon	8.44 ± 9.22	0.363		
Left colon	−1.73 ± 11.5	0.880		
Macroscopic type				
LST-NG	31.40 ± 13.69	0.24		
LST-G	31.35 ± 12.91	0.27		
Others	28.60 ± 8.80	0.456		
Histology				
Adenocarcinoma	39.36 ± 13.51	0.005	29.00 ± 13.10	0.06
Adenoma	14.39 ± 7.70	0.065	20.09 ± 12.93	0.12
Non lifting sign	3.42 ± 9.49	0.719		

ESD: endoscopic submucosal dissection; LST-NG: laterally spreading tumors-nongranular type; LST-G: laterally spreading tumors-granular type.

**Table 4:** Previous reports of colorectal ESD.

Author, year	*n*	En bloc resection	R0 resection	Perforation
Saito et al., 2007 [13]	200	84%	83%	5%
Fujishiro et al., 2007 [14]	200	91.5%	71%	6%
Saito et al., 2010 [3]	1111	88%	n.a.	5.2%
Nishiyama et al., 2010 [15]	296	89.2%	79.1%	8.1%
Tanaka et al., 2010 [16]	8303	83.8%	n.a.	4.8%
Our study	92	90.2%	89.1%	12%

ESD: endoscopic submucosal dissection; *n*: number; n.a.: not available.

## References

[1] Liou JM, Lin JT, Huang SP (2007). Screening for colorectal cancer in average-risk Chinese population using a mixed strategy with sigmoidoscopy and colonoscopy. *Diseases of the Colon and Rectum*.

[2] Cunningham D, Atkin W, Lenz HJ (2010). Colorectal cancer. *The Lancet*.

[3] Saito Y, Uraoka T, Yamaguchi Y (2010). A prospective, multicenter study of 1111 colorectal endoscopic submucosal dissections (with video). *Gastrointestinal Endoscopy*.

[4] Tanaka S, Oka S, Kaneko I (2007). Endoscopic submucosal dissection for colorectal neoplasia: possibility of standardization. *Gastrointestinal Endoscopy*.

[5] Taku K, Sano Y, Fu KI (2007). Iatrogenic perforation associated with therapeutic colonoscopy: a multicenter study in Japan. *Journal of Gastroenterology and Hepatology*.

[6] Yoshida N, Yagi N, Naito Y, Yoshikawa T (2010). Safe procedure in endoscopic submucosal dissection for colorectal tumors focused on preventing complications. *World Journal of Gastroenterology*.

[7] Lee DS, Jeon SW, Park SY (2010). The feasibility of endoscopic submucosal dissection for rectal carcinoid tumors: comparison with endoscopic mucosal resection. *Endoscopy*.

[8] (2002). The Paris endoscopic classification of superficial neoplastic lesions: esophagus, stomach, and colon. *Gastrointestinal Endoscopy*.

[9] Kudo SE, Tamura S, Nakajima T, Yamano HO, Kusaka H, Watanabe H (1996). Diagnosis of colorectal tumorous lesions by magnifying endoscopy. *Gastrointestinal Endoscopy*.

[10] Uraoka T, Saito Y, Matsuda T (2006). Endoscopic indications for endoscopic mucosal resection of laterally spreading tumours in the colorectum. *Gut*.

[11] Kitajima K, Fujimori T, Fuji S (2004). Correlations between lymph node metastasis and depth of submucosal invasion in submucosal invasive colorectal carcinoma: a Japanese collaborative study. *Journal of Gastroenterology*.

[12] Ueno H, Mochizuki H, Hashiguchi Y (2004). Risk factors for an adverse outcome in early invasive colorectal carcinoma. *Gastroenterology*.

[13] Saito Y, Uraoka T, Matsuda T (2007). Endoscopic treatment of large superficial colorectal tumors: a case series of 200 endoscopic submucosal dissections (with video){A figure is presented}. *Gastrointestinal Endoscopy*.

[14] Fujishiro M, Yahagi N, Kakushima N (2007). Outcomes of endoscopic submucosal dissection for colorectal epithelial neoplasms in 200 consecutive cases. *Clinical Gastroenterology and Hepatology*.

[15] Nishiyama H, Isomoto H, Yamaguchi N (2010). Endoscopic submucosal dissection for colorectal epithelial neoplasms. *Diseases of the Colon and Rectum*.

[16] Tanaka S, Tamegai Y, Tsuda S, Saito Y, Yahagi N, Yamano HO (2010). Multicenter questionnaire survey on the current situation of colorectal endoscopic submucosal dissection in Japan. *Digestive Endoscopy*.

[17] Hotta K, Saito Y, Matsuda T, Shinohara T, Oyama T (2010). Local recurrence and surveillance after endoscopic resection of large colorectal tumors. *Digestive Endoscopy*.

[18] Saito Y, Fukuzawa M, Matsuda T (2010). Clinical outcome of endoscopic submucosal dissection versus endoscopic mucosal resection of large colorectal tumors as determined by curative resection. *Surgical Endoscopy and Other Interventional Techniques*.

[19] Axon A, Diebold MD, Fujino M (2005). Update on the Paris classification of superficial neoplastic lesions in the digestive tract. *Endoscopy*.

[20] Matsuda T, Fujii T, Saito Y (2008). Efficacy of the invasive/non-invasive pattern by magnifying chromoendoscopy to estimate the depth of invasion of early colorectal neoplasms. *American Journal of Gastroenterology*.

[21] Park HW, Byeon JS, Park YS (2010). Endoscopic submucosal dissection for treatment of rectal carcinoid tumors. *Gastrointestinal Endoscopy*.

[22] Kim HH, Park SJ, Lee SH (2012). Efficacy of endoscopic submucosal resection with a ligation device for removing small rectal carcinoid tumor compared with endoscopic mucosal resection: analysis of 100 cases. *Digestive Endoscopy*.

[23] Hotta K, Oyama T, Shinohara T (2010). Learning curve for endoscopic submucosal dissection of large colorectal tumors. *Digestive Endoscopy*.

